# Evolution of endoscopic vacuum therapy for upper gastrointestinal leakage over a 10-year period: a quality improvement study

**DOI:** 10.1007/s00464-022-09400-w

**Published:** 2022-07-19

**Authors:** Stanislaus Reimer, Florian Seyfried, Sven Flemming, Markus Brand, Alexander Weich, Anna Widder, Lars Plaßmeier, Peter Kraus, Anna Döring, Ilona Hering, Mohammed K. Hankir, Alexander Meining, Christoph-Thomas Germer, Johan F. Lock, Kaja Groneberg

**Affiliations:** 1 Department of Gastroenterology, University Hospital of Würzburg, Würzburg, Germany; 2 Department of General-, Visceral-, Transplant-, Vascular- and Pediatric Surgery, University Hospital of Würzburg, Würzburg, Germany; 3 Department of General, Visceral, Transplantation, Vascular and Pediatric Surgery, Center of Operative Medicine (ZOM), University Hospital of Würzburg, Würzburg, Germany

**Keywords:** Anastomotic leak, Gastrointestinal perforation, Esophageal perforation, Endoluminal, Vacuum-assisted closure, Negative pressure

## Abstract

**Background:**

Endoscopic vacuum therapy (EVT) is an effective treatment option for leakage of the upper gastrointestinal (UGI) tract. The aim of this study was to evaluate the clinical impact of quality improvements in EVT management on patients’ outcome.

**Methods:**

All patients treated by EVT at our center during 2012–2021 were divided into two consecutive and equal-sized cohorts (period 1 vs. period 2). Over time several quality improvement strategies were implemented including the earlier diagnosis and EVT treatment and technical optimization of endoscopy. The primary endpoint was defined as the composite score MTL30 (mortality, transfer, length-of-stay > 30 days). Secondary endpoints included EVT efficacy, complications, in-hospital mortality, length-of-stay (LOS) and nutrition status at discharge.

**Results:**

A total of 156 patients were analyzed. During the latter period the primary endpoint MTL30 decreased from 60.8 to 39.0% (*P* = .006). EVT efficacy increased from 80 to 91% (*P* = .049). Further, the need for additional procedures for leakage management decreased from 49.9 to 29.9% (*P* = .013) and reoperations became less frequent (38.0% vs.15.6%; *P* = .001). The duration of leakage therapy and LOS were shortened from 25 to 14 days (*P* = .003) and 38 days to 25 days (*P* = .006), respectively. Morbidity (as determined by the comprehensive complication index) decreased from 54.6 to 46.5 (*P* = .034). More patients could be discharged on oral nutrition (70.9% vs. 84.4%, *P* = .043).

**Conclusions:**

Our experience confirms the efficacy of EVT for the successful management of UGI leakage. Our quality improvement analysis demonstrates significant changes in EVT management resulting in accelerated recovery, fewer complications and improved functional outcome.

**Supplementary Information:**

The online version contains supplementary material available at 10.1007/s00464-022-09400-w.

Leaks and perforations of the upper gastrointestinal tract (UGI) are life-threatening conditions with a multifactorial etiology [1]. A growing body of evidence indicates that the endoscopic management of these transmural defects has evolved considerably over the last decade in particular [2]. Endoscopic negative pressure or vacuum therapy (EVT) of the UGI was originally introduced to overcome the high mortality rates associated with surgical repair [3] as well as the problems that come with self-expanding metal stent (SEMS) therapy [4]. Due to its high rates of success even in extreme cases, EVT has become an established therapeutic option, especially for the management of postoperative anastomotic leaks after oncological UGI surgery as it offers considerable advantages over other endoscopic and surgical interventions [5]. Consequently, EVT is now routinely performed by many visceral medical centers around the globe [6–8].

The advantages of EVT are numerous and have been reported elsewhere [9]. However, most of the evidence in favor of EVT comes from heterogeneous studies involving a limited number of patients or case reports [10]. Thus, no detailed recommendations of when or how to apply EVT have yet been made available.

The aim of this study was to evaluate the local quality improvements in EVT of transmural UGI defects at our tertiary center after its first application in 2012. We hypothesized that analogous to other endoscopic interventions [11], the greater experience with EVT in conjunction with adjustments in institutional factors, overall patient management and technical details positively impacts its overall efficacy and outcomes of UGI leakage.

## Methods

This quality improvement study covered a 10-year period (2012–2021) and was retrospectively conducted in a 1500-bed tertiary center (University Hospital Würzburg, Germany). The manuscript was prepared according to the Standards for Quality Improvement Reporting Excellence (SQUIRE) [12].

### Study design and ethics

The impact of changes in the clinical and endoscopic management of UGI leaks managed by EVT were evaluated for quality improvement through a cohort analysis. All patients with UGI defects treated by EVT at our institution were divided into two consecutive and equal-sized patient cohorts (period 1 vs. 2). Since the first application of EVT at our center in 2012, the local clinical management of UGI leakage has undergone several changes including personnel and qualified endoscopists, the diagnosis and management of leakages, and technical endoscopic aspects (see details in Table 1 and Supplementary Methods section). The study did not comprise additional procedures or examinations and was based on clinical data available from the hospital information management system. From 2015, all cases were prospectively collected in a standardized database. The study was approved by the local ethics review board (Ethics committee, Würzburg University) in 2015.

**Table 1:** Local quality improvements aspects in leakage therapy

Characteristic	Period 1	Period 2
Staff and organization management		
Endoscopic faculties involved, *n*	2	1
Leading coordinator for EVT	No	Yes
Broad endoscopic experience (including clipping, stenting, necrosectomy, hemostasis) requested for performance of EVT	No	Yes
No. of “on-call” endoscopists, *n*	15	4
Diagnosis of leakage		
Indication for postoperative leakage diagnostics	Clinical deterioration	Any deviation from normal course
Primary diagnostic technique	Barium meal CT	Endoscopy
Timing of endoscopy	Within 24 h	Within 6 h
Primary leakage therapy	Surgery, stent or EVT	EVT
Endoscopic techniques and application of negative pressure		
Protective intubation for endoscopy	Routinely in sepsis	Only in respiratory distress
Removal of foreign material (clips, staples, etc.)	Occasionally	Routinely
Endoscopic necrosectomy and tissue debridement	Occasionally	Routinely
Application of OTSG Xcavator (Ovesco)	No	Yes
Simultaneous use of multiple sponges and pumps	No	If required
Feeding tube along the same route with sponge	Yes	No
Endoscopic emptying of organ distal to leak	Occasionally	Routinely
Flushing sponge before removal	Routinely	None
Tube diameter	Randomly	Standard 14F
No of systems for application of negative pressure (*n*)	4	1
Use of additive endoscopic leakage closure to shorten EVT (OTSC, Ovesco))	Occasionally	Routinely

*EVT* endoscopic vacuum therapy; *OTSG* over-the-scope grasper; *OTSC* over-the-scope-clip

### Endoscopic vacuum therapy

This technique requires a flexible endoscope to place an open-pored polyurethane sponge into the cavity behind the leak (intracavitary) or within the intestinal lumen (intraluminal) [13]. The sponge was connected by a nasogastric tube to a negative pressure system. An intracavitary sponge was usually adopted for accessible extraluminal cavities; an intraluminal sponge was generally preferred for defects with diffuse local inflammation or shallow cavities. The sponge was changed regularly every 3–4 days [14]. EVT was terminated when stable granulation tissue covered and no signs of necrosis or leakage were present.

**Fig. 1:**
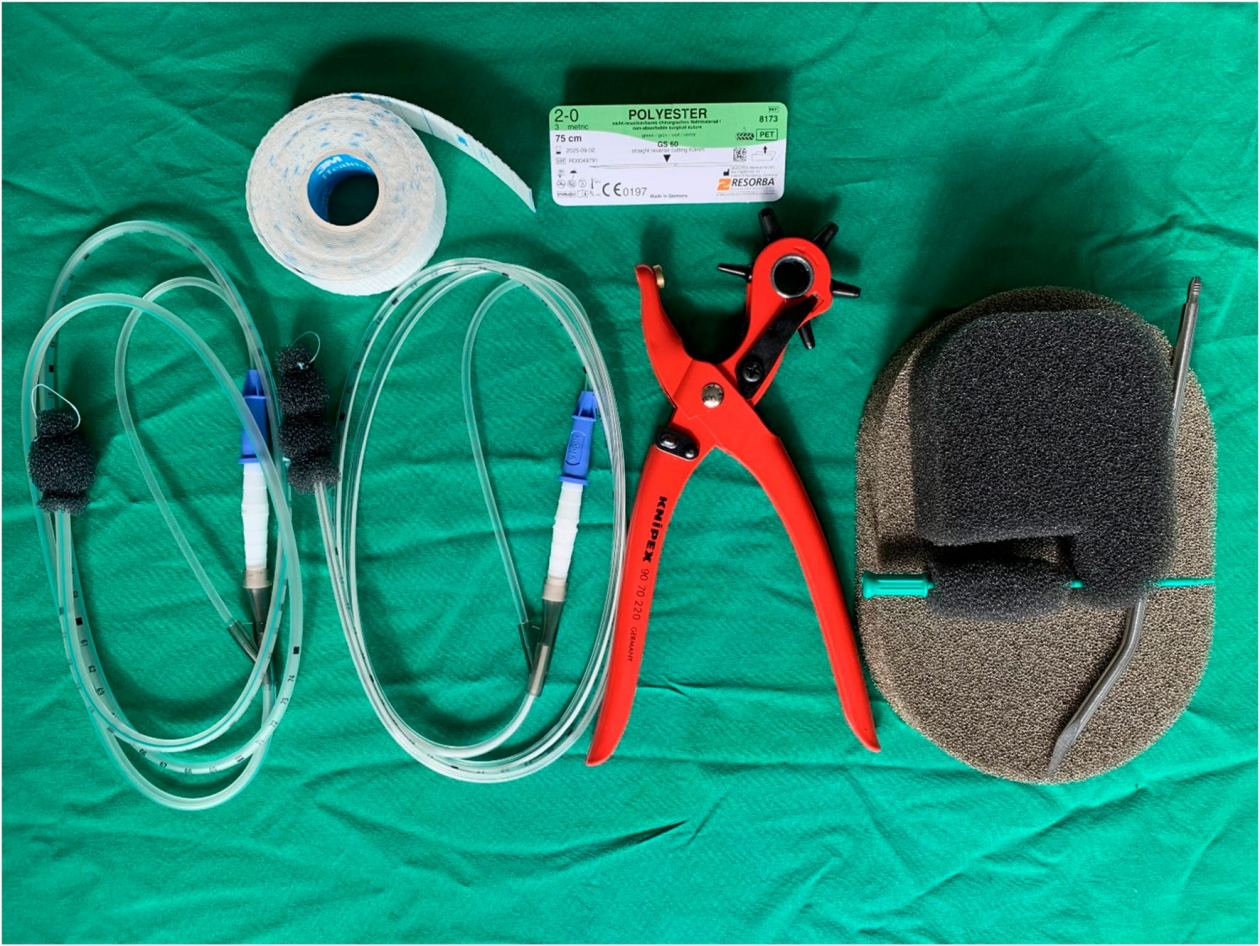
Materials applied for EVT. EsoSPONGE® attached to a 14F-tube, tape for tube-fixation, EndoSPONGE® attached to a 14F-tube, suture material, pliers for additional holes, Granufoam silver Dressing, Invia Foam Dressing, 16F-metal tip for tube insertion

The vast majority of reported EVT applications at our center were carried out with modified commercially available open-pore polyurethane foam drains that are approved as medical devices for treatment of the esophagus and rectum (EndoSPONGE® and EsoSPONGE®, both B. Braun Melsungen AG, Melsungen, Germany). The modification included removal of the sponge from the original draining tube at the proximal end. The sponge was then carefully cleaned and attached to a 14F gastric tube with 10 perforations on both sides over a length of 6 cm (Vygon, Ecouen, France) with several stitches. A 16F tube was used to drain particularly viscous mucus and a 12F probe was used for angled approaches, smaller cavities, less compliant patients and duodenal lesions. The tip of the tube was snipped off after the sponge was attached to the probe and about 5–7 mm was pulled back into the sponge so that the sponge tip was soft. For localized tissue defects, care was taken to ensure that the suction effect was focused on the defect so that it closed and did not spread to surrounding tissue for avoidance (of stricture formation). In our experience the number and arrangement of the holes on the gastric tube should be limited and restricted to the area carrying the sponge. Therefore, the tube was shortened and additional holes were created on the probe using pliers when necessary (Knipex-Werk C. Gustav Putsch KG, Wuppertal Germany). EndoSPONGE® was used mainly during the first period. In total, < 5% of treatments required a sponge longer than 5 cm (V.A.C. Granufoam Dresssing, 3 M, San Antonio, USA or Invia Foam Dressing, Medela, Baar, Switzerland were used). Figure 1 shows tools as applied.

Foreign body forceps (Rat Tooth Forceps, Endo-Flex GmbH, Voerde, Germany) were applied for endoscopic sponge placement. Standard biopsy forceps and foreign body forceps (Radial Jaw 4, standard capacity, Radial Jaw 4, Jumbo, Boston Scientific, Marlborough, USA and Rat Tooth Forceps, Endo-Flex GmbH, Voerde, Germany) were used for necrosectomy and cleaning the defect margins. In addition an over-the-scope grasper (OTSG, Xcavator, Ovesco AG, Germany) was occasionally used if extended necrosectomy was necessary. A biliary cytology brush (Cytomax II double lumen, cytology brush, Cook medical, Bloomington, USA) was used to refresh the fistula opening and canal if necessary.

### Endpoints

The primary endpoint was defined as the quality indicator MTL30 (mortality, transfer, length of stay > 30 days). This composite endpoint is a well-defined surgical quality indicator, integrating both the occurrence of negative outcomes (in particular patient death) as well as prolonged recovery or additional complications with long hospitalization [15, 16]. The secondary endpoints included the clinical efficacy of EVT, additional procedures during leakage therapy, local complications during EVT, tracheo- or bronchoesophageal fistula (TBF), in-hospital mortality, length-of-stay and nutrition status at discharge.

Detailed description of all collected variables are provided in supplementary methods section.

### Statistical analysis

All statistical analyses were performed using IBM SPSS Statistics 26 (International Business Machines Corporation, Armonk, NY). Descriptive data are reported as means with standard deviations, unless otherwise stated. Comparisons between the analyzed cohorts were performed using Chi-square, Fisher’s exact, Mann–Whitney *U* tests or one-way analysis of variance, in accordance with data scale and distribution. The time-intervals were compared by Kaplan–Meier analysis with log rank test. The level of statistical significance was 0.05 (two-sided).

## Results

### Study population and indications for EVT

Detailed baseline patient characteristics are provided in Table 2. A total of 156 patients have underwent EVT at our institution since 2012. The main indication was anastomotic leakage in over 64% of EVT cases. The majority of patients requiring EVT suffered from malignant tumors (56%). The total numbers of surgical procedures during 2012–2021 and those with postoperative leakages treated using EVT are provided in Supplementary Table 1. The incidence of postoperative leakage was 10-times higher after oncological resections in comparison to procedures for benign disease (9.8% vs. 0.9%). No significant changes concerning the underlying disease or etiology of leakage, nor the comorbidity or time-point of diagnosis were observed between the study periods. However, the mean age of patients increased from 55.5 to 61.9 years (*P* = 0.005) and the percentage of patients with previous neoadjuvant tumor therapy increased from 31.6 to 49.4% (*P* = 0.001).

**Table 2:** Patient characteristics and indications for EVT

Characteristic	Patients, No. (%)			*P* value
	Total (*n* = 156)	Period 1 (*n* = 79)	Period 2 (*n* = 77)	
Sex ratio, No. (M:F)	106: 50	54: 25	52: 25	1
Age, mean (SD), y	58.7 (14.3)	55.5 (14.7)	61.9 (13.2)	.005*
BMI, mean (SD) (kg/m^2^)	28.0 (8.4)	29.1 (8.5)	26.8 (8.1)	.09
Charlson comorbidity index, mean (SD)	3.9 (2.6)	3.7 (2.4)	4.1 (2.7)	.30
ASA classification ≥ III	98 (63.2)	44 (56.5)	54 (70.1)	.057
Benign disease	68 (43.6)	35 (44.3)	33 (42.9)	.86
Malignant tumor	88 (56.4)	44 (55.7)	44 (57.1)	
Neoadjuvant therapy	63 (40.4)	25 (31.6)	38 (49.4)	.001*
Type of leakage				.52
Primary perforation	10 (6.4)	5 (6.3)	5 (6.5)	
Postoperative preventive^†^	15 (9.6)	8 (10.1)	7 (9.1)	
Iatrogenic perforation^‡^	31 (19.8)	22 (27.8)	9 (11.7)	
Anastomotic insufficiency	100 (64.1)	44 (55.7)	56 (72.7)	
Esophago-gastrostomy	44 (44.0)	19 (43.2)	25 (44.6)	.106
Esophago-jejunostomy	31 (31.0)	11 (25.0)	20 (35.7)	
Gastro-jejunostomy	21 (21.0)	10 (22.7)	11 (19.6)	
Other	4 (4.0)	4 (9.1)	0	
Previous surgery	142 (91.0)	70 (88.6)	72 (93.5)	.57
Oncological surgery UGI	71 (57.3)	35 (58.3)	36 (56.3)	
Other UGI	50 (41.3)	22 (38.6)	28 (43.8)	
Other	16 (10.3)	12 (15.2)	4 (5.2)	
Interval from surgery to diagnosis of leakage, mean (95%CI), d	9.7 (8.5–11.1)	11.0 (8.9–13.1)	8.5 (6.9–11.1)	.063

Values are *n* (%) unless otherwise indicated

*EVT* endoscopic vacuum therapy; *SD* standard deviation; *95%CI* 95% confidence interval

^†^Intraoperative endoscopy and start of EVT during surgery due to expected high risk of anastomotic leakage. ^‡^Including leakages after endoscopic procedures, postoperative perforations and suture line leakages, except anastomotic leakage

*P < .05

### Changes during leakage therapy

Detailed changes in leakage therapy are provided in Table 3. The number of leakage interventions and procedures prior to EVT decreased from 1.4 to 0.4 (*P* = 0.014) and the interval from diagnosis of leakage until the start of EVT decreased from 7.2 days to 0.3 days (*P* = 0.016). There were no significant differences in leakage diameter (11.8 mm vs. 8.9 mm) or the incidence of sepsis (39% vs. 37%) at the start of EVT. The rate of ventilated patients at the start of EVT significantly decreased (43% vs. 23%, *P* = 0.009).

**Table 3:** Changes during leakage therapy

Characteristic	Patients, No. (%)			*P* value
	Total (*n* = 156)	Period 1 (*n* = 79)	Period 2 (*n* = 77)	
No. of previous procedures for leakage therapy before start of EVT, mean (95%CI)	0.9 (0.5–1.3)	1.4 (0.7–2.2)	0.4 (0.1–0.8)	.014*
Interval from diagnosis of leakage until start of EVT, mean (95%CI), d	3.8 (1.0–6.6)	7.2 (1.6–12.7)	0.3 (0.1–0.7)	.016*
Leakage diameter, mean (95%CI), mm	10.4 (8.7–12.1)	11.8 (9.2–4.5)	8.9 (6.9–1.1)	.09
Sepsis at start of EVT	59 (38.1)	31 (39.2)	28 (36.8)	.76
Mechanical ventilation at start of EVT	52 (33.3)	34 (43.0)	18 (23.4)	.009*
Duration of leakage therapy, median (quartiles), d	17 (8–33)	25 (11–39)	14 (7–28)	.003*
Sponge changes, median (quartiles)	4 (2–8)	4 (2–7)	5 (2–8)	.20
Management on general ward	66 (42.3)	31 (39.2)	35 (45.5)	.63
Total parenteral nutrition during EVT	97 (62.2)	49 (62.0)	48 (62.3)	.97
Local complications during EVT	28 (17.9)	16 (20.3)	12 (15.6)	.29
Stenosis/stricture	17 (10.9)	8 (10.1)	9 (11.7)	.75
Acute bleeding	3 (1.9)	1 (1.3)	2 (2.6)	.54
TBF	9 (5.8)	8 (10.3)	1 (1.3)	.017*
Recurrent sepsis	26 (16.8)	16 (20.5)	10 (13.0)	.15
Additional procedures during EVT	62 (39.7)	39 (49.4)	23 (29.9)	.013*
Reoperation	42 (26.9)	30 (38.0)	12 (15.6)	.001*
Pleural decortication	16 (10.3)	14 (17.7)	2 (2.6)	.004*
Percutaneous drainage	53 (34.0)	30 (38.0)	23 (29.9)	.28
Additional procedures after EVT	32 (20.5)	14 (17.7)	18 (23.4)	.38
Endoscopic clip	19 (12.2)	3 (3.8)	16 (20.8)	.001*
SEMS	13 (8.3)	11 (13.9)	2 (2.6)	.010*

Values are *n* (%) unless otherwise indicated

*EVT* endoscopic vacuum therapy; *95%CI* 95% confidence interval; *TBF* tracheo- or bronchoesophageal fistula; *SEMS*, self-expanding metal stent

*P < .05

**Fig. 2:**
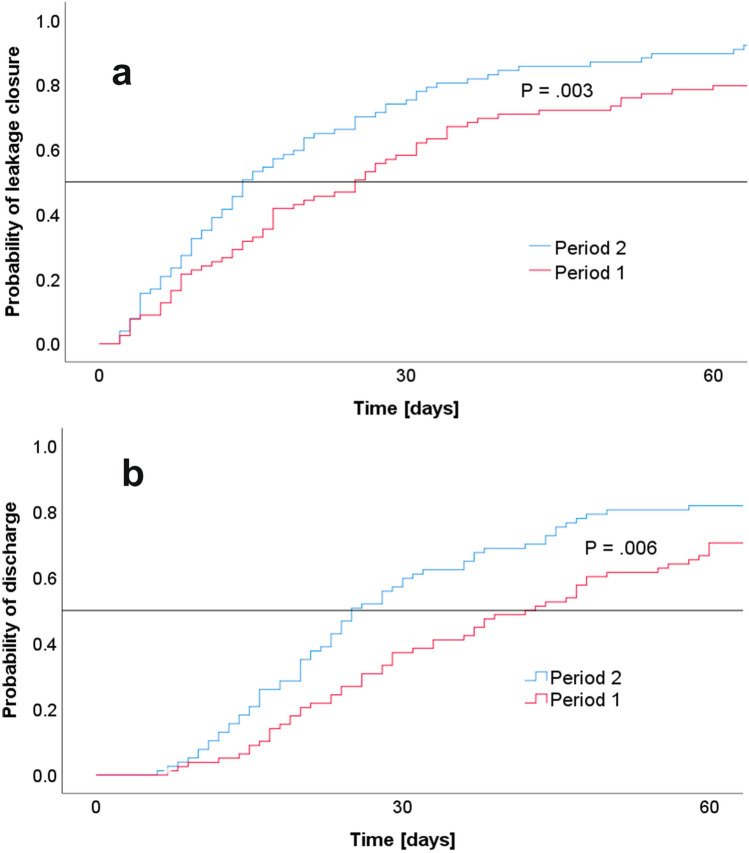
Time changes in leakage management. Kaplan–Meier analysis with log rank test. **a** Duration of leakage therapy. **b** Length-of-stay

However, only a minority of patients were managed on a general ward (39% vs. 37%). Most patients received total parenteral nutrition during EVT (62% in both periods). No changes in the development of anastomotic stenosis (10% vs. 12%) or EVT associated GI bleeding (1.3% vs. 2.6%) were recorded. The risk of TBF was reduced from 10 to 1% (*P* = 0.017). Additional procedures for management of leakage or local infection control decreased from 49.9 to 29.9% (*P* = 0.013). In particular, the need for thoracotomy to manage pleural empyema decreased from 17.7 to 2.6% (*P* = 0.004). While some patients received SEMS (*n* = 11) upon completion of EVT by 2016, an increasing number of patients received OTSC (*n* = 16) upon completion of EVT since 2019. Finally, the duration of leakage therapy was shortened from a median of 25 days to 14 days (*P* = 0.003; see Fig. 2A).

### Efficacy of EVT

Details of EVT efficacy are provided in Table 4. Improvement of leakages during EVT was observed in most patients from the first application. Eight of the first ten patients treated by EVT at our institution showed improvement and complete leakage resolution. Nevertheless, the clinical success rate defined as full resolution of leakage during EVT significantly increased from 79.7 to 90.9% (*P* = 0.049). More than 70% of patients were managed without any additional surgical procedure or percutaneous drainage in period 2. Preventive postoperative EVT (mean duration of 7 days) was successful in 13 out of 15 (86.6%) of patients who did not develop anastomotic leakage during follow-up. Three of these patients received EVT simultaneously during early surgical revision due to leakage or ischemic gastric conduit. The remaining patients received preventative EVT when a postoperative diagnostic gastroscopy for suspected leakage was performed but no evident leakage was found. In these cases of uncertainty, the endoscopists chose to start preventative EVT.

**Table 4:** Patients’ outcome

Characteristic	Patients, No. (%)			*P* value
	Total (*n* = 156)	Period 1 (*n* = 79)	Period 2 (*n* = 77)	
MTL30	78 (50.0)	48 (60.8)	30 (39.0)	.006*
In-hospital mortality	10 (6.4)	7 (8.9)	3 (3.9)	.17
Efficacy of EVT				
Improvement of leakage	140 (89.7)	68 (86.1)	72 (93.5)	.10
Resolution of leakage	133 (85.3)	63 (79.7)	70 (90.9)	.049*
Resolution without additional procedures during or after EVT	94 (60.3)	40 (50.6)	54 (70.1)	.013*
Failure-to-cure†	18 (11.5)	12 (15.2)	6 (7.8)	.12
Clavien–Dindo ≥ grade IIIb	74 (47.4)	42 (53.2)	32 (41.6)	.09
CCI, mean (95%CI)	50.6 (46.8–54.3)	54.6 (49.3–59.9)	46.5 (41.3–51.8)	.034*
Length-of-stay, median (quartiles), d	30 (20—53)	38 (23–60)	25 (16–44)	.006*
Oral nutrition on discharge	121 (77.6)	56 (70.9)	65 (84.4)	.043*

Values are *n* (%) unless otherwise indicated

*MTL30* in-hospital-mortality or length-of-stay > 30 days; *CCI* comprehensive complication index;

^†^Conversion to surgical therapy due to deteriorating leakage during EVT or death

*P < .05

In addition, the rate of failure-to-cure decreased by 50% (15.2 vs. 7.8%) but this did not attain statistical significance. In these 18 (11.5%) patients, leakage therapy was not successful by EVT and the leakage was managed by surgery (8 patients) or patients died during hospitalization (10 patients; individual details are provided in Supplementary Table 2). An additional two patients died despite successful leakage therapy, one due to pneumonia (18 days after resolution of the leakage), and another due to acute pulmonary embolism (2 days after resolution of the leakage). The remaining 8 patients died from sepsis and further complications during leakage therapy.

### Patient outcome

Details on patient outcomes are provided in Table 4. The primary endpoint MTL30 decreased from 60.8 to 39.0% (*P* = 0.006). The in-hospital mortality decreased by over 50% (8.9% vs. 3.9%) but this did not attain statistical significance. The CCI decreased by 8 points (*P* = 0.034) and the LOS was 13 days shorter in median (38 days vs. 25 days; *P* = 0.006; see Fig. 2B). At discharge from hospital, the majority of patients achieved sufficient oral nutrition with increasing success rates (70.9% vs. 84.4%; *P* = 0.043).

## Discussion

Our local experience during a recent, 10-year period confirms the efficacy of EVT in UGI leakage management with clinical success rates > 85%. However the quality improvement analysis demonstrates significant changes in leakage management. In particular, our analysis supports the application of EVT as a first-line therapy and low-threshold indication for postoperative endoscopy and the early start of EVT with aggressive debridement. These changes significantly improved the efficacy of EVT as reflected by the composite endpoint MTL30.

We report the largest patient cohort treated with EVT to date. While our cohort was a heterogeneous one, the majority of patients suffered from postoperative anastomotic insufficiency after oncological UGI surgery similar to most previous reports with more homogeneous cohorts [2]. We chose to focus on the management of UGI leakage in this analysis and not on its etiology. Nevertheless, the comparison of patient and leakage characteristics between the study groups demonstrated no relevant differences that could have biased the results of the quality improvement analysis. On the contrary, patients in period 2 were significantly older and had more frequently received neoadjuvant tumor therapy.

The comparison of leakage management between periods 1 and 2 highlight the impact of the local quality improvement intervention: The lower threshold for postoperative endoscopy instead of radiologic imaging lead to a trend towards earlier postoperative diagnosis of leakage (8 vs. 11 days). This early time-point in period 2 corresponds well to the 8 days reported in recent studies for postoperative leakage after oncologic UGI surgery [17, 18]. Notably, no changes in leakage diameter or presence of sepsis on diagnosis of leakage were observed in our analysis. Nevertheless, there might be a relevant bias due to the heterogeneity of leakage etiology with a relevant number of patients with primary perforations or other non-UGI procedures. Therefore, further subgroup analysis focusing on postoperative leakage after oncological UGI surgery are required to confirm positive clinical effects from early endoscopic leakage diagnosis.

The most relevant change in management concerned the interval from diagnosis of leakage until start of EVT. While patients in period 1 frequently either underwent revisional surgery, received SEMS or a primary conservative approach with gastric tube only, nearly all patients in period 2 directly received EVT upon diagnostic endoscopy. Thus, the duration of leakage until EVT was significantly shortened and the number of leakage interventions prior to EVT were reduced to a minimum. An analogous approach was reported from Heidelberg University Hospital, where postoperative leakage management was switched to primary EVT in 2015 [17]. The earlier diagnosis of leakages and the abandonment of revisional surgery reduced the incidence of mechanical ventilation by 50% in our analysis, which likely prevents additional pulmonary complications and long-term ICU stay. In addition, EVT provided effective infection control as the number of additional procedures to treat infection could be significantly reduced in period 2. In particular, pleural empyema requiring surgical thoracic debridement with pleural decortication became extremely rare.

The changes in leakage management enabled a significant shortening of leakage therapy to a median of 9 days. The duration of leakage therapy in period 2 was thus shorter than in most previous reports [13]. Since EVT can become lengthy, especially in critically ill patients, several patients were switched to stent therapy after improvement of leakage in period 1. This was however abandoned since it required additional endoscopic procedures and the stents caused further problems, in particular dislocation along with persistent leakage or fistula creation. Therefore, we decided to shorten leakage therapy by conversion to clip closure by OTSC (Ovesco AG, Germany) after profound improvement during EVT, resulting in decreased duration of leakage therapy and high success rates.

The efficacy of EVT in period 1 was already in line with previous reports with an almost 80% success rate. The earlier diagnosis and treatment along with technical changes in endoscopic management lead to an increased success rate of 91% in period 2. This high efficacy is consistent with data derived from smaller and more distinctive series [2, 18]. Unsuccessful EVT, so called “failure-to-cure”, was recorded in fewer patients in period 2 (8 vs. 15%) but did not attain statistical significance due to sample numbers. Concerning local complications from EVT, these occurred at a comparable incidence of 15–20% which is in accordance with the literature [5, 18]. The majority of cases with EVT complications developed local stenosis. However, stenosis occurs equally or even more often during stent therapy [18]. According to the available literature and our own experience, these can usually be treated successfully with pneumatic dilatation [19]. The most severe and frequently fatal complication of TBF was reduced by 90% in period 2 despite a higher frequency of thoracic esophageal resection in that group. We note that we interpret TBF as a sequel of ineffective primary leakage management rather than as a complication of EVT. Importantly, the available literature and our data both suggest that the presence of TBF is one of the very few situations that preclude successful treatment with EVT [20].

In our experience, there are only very few clinical situations that preclude successful EVT. We found that patients with a bronchial or tracheal fistula are usually not eligible candidates for EVT. Another problem can be patient compliance and the inconvenience of a nasoesophageal tube. Patients require individual and clear communication emphasizing the medical importance of EVT to prevent further sequelae from the leakage. In addition, medical personnel involved in patients receiving EVT should receive special training to avoid sponge dislocation while enabling normal patient mobilization and physical therapy. Only 3 out of 156 patients (1.9%) did not tolerate EVT. Thus, we chose to perform temporary deep sedation with intubation and ventilation. This aggressive EVT strategy warrants careful consideration of alternative surgical therapies and their estimated success rates. Another problem can be limited endoscopic access, particularly in the distant duodenal position. We previously published a case report in which jejunostomy was created to provide endoscopic access to the duodenal leakage resulting in leakage resolution [21]. Finally, in some cases where leakage occurs with additional problems such as local ischemia or severe strictures, EVT might not be the ultimate solution.

We also used EVT in some patients to achieve local infection control prior to reconstructive surgery. This concept was for example applied in an externally assigned complex case with cervical esophageal perforation [22]. In these cases EVT functioned as a part of a visceral medical complex under interdisciplinary treatment but not as a standalone therapy [22].We routinely evaluate all cases (especially complex ones) in a close-knit interdisciplinary manner to identify the best available therapy alternatives according to each patient’s individual course. Thus, therapy adjustments (both endoscopic and surgical) could be carried out in a timely and tailored manner.

Concerning patients’ outcome after leakage, our local quality improvement intervention shortened the overall hospital stay by a median of 13 days. The mortality could be reduced to 3.9%—although this result was not significant due to the small sample size. It is important to note that only a part of the mortality was causally attributable to a “failure to cure” the leakage, but rather to additive or simultaneous complications (e.g. pulmonary embolism, aspiration, etc.). In the literature, the leak-associated mortality for esophagectomy ranges between 7.2 and 35%, so our results suggest a very successful leakage management [13, 23]. Overall, the recent improvements are reflected in a relevant MTL-30 reduction, a ‘composite score’ established for a large number of visceral surgical interventions and a quality indicator used by the German Society for General and Visceral Surgery [15, 16]. Accordingly, CCI, which is another validated quality indicator, also decreased significantly.

With respect to enteral nutrition of patients during EVT, we chose to avoid transesophageal feeding tubes since they obstruct leakages from direct contact with the sponge and might increase the volume of exposure to the suction system. Patients with thoracic esophageal resection routinely receive percutaneous feeding tubes and thus enteral nutrition was continued during EVT. The other patients received total parenteral nutrition during EVT. Although we acknowledge that enteral nutrition is highly favorable, our experience confirms that strictly focusing on optimal leakage management is justified. The good functional outcome reflected by full oral intake at discharge in almost 85% of patients in period 2 supports this strategy.

To our knowledge, we provide the largest number of EVT cases with detailed data reporting including the presentation of the ‘failure to cure’ cases. This is the first study describing in detail institutional and technical improvements with a relevant impact on the outcome. A weakness of our study is its retrospective design, although from 2015 all cases were prospectively collected in a standardized database. We chose a dichotomous comparison of our early and late patients by forming two equal-sized patient samples. Even if many details of the technical optimizations were not implemented on a set date, but rather continuously, the relevant changes mentioned above took place at this point in time. The comparatively high number of cases in the two groups provided the necessary statistical power for subgroup analyses. It should be mentioned in particular that the entities and configurations of the leaks were not different in the two groups. We acknowledge that a precise definition of the leaks, as described by the Esophagectomy Complications Consensus Group for oncological esophageal resections [24], would generally be desirable to achieve better comparability and to provide clearer therapeutic recommendations in the future. However, currently available recommendations do not consider the availability of EVT therapy as a possible option [25]. It cannot be ruled out that, especially during period 2, due to our active and progressive treatment strategy, a proportion of patients with clinically covert leakage might have healed with purely conservative treatment and may thus have been over-treated using EVT. Nevertheless, the potential of preventive primary EVT in high risk patients for UGI anastomotic leakage warrants further examination.

In summary, the implementation of EVT represents a revolutionary improvement in the management of UGI leakages. With the earliest possible initiation of therapy and appropriate expertise, good therapeutic success can usually be achieved quickly, and fistula formation and further septic decompensation can be prevented in the majority of cases. Our data show that early EVT treatment together with optimization of institutional and technical aspects can significantly improve patient outcome.

## Supplementary Information

Below is the link to the electronic supplementary material.

Supplementary file1 (DOCX 66 kb)
