# Supplementary material for: Evolution of endoscopic vacuum therapy for upper gastrointestinal leakage over a 10-year period: a quality improvement study
Source: Surg Endosc. 2022 Jul 19;36(12):9169–78. doi: 10.1007/s00464-022-09400-w (PMC9652162; doi:10.1007/s00464-022-09400-w)
Supplement: Supplementary file 1 — Supplementary file1 (DOCX 66 kb) [file 464_2022_9400_MOESM1_ESM.docx]

**SUPPLEMENTARY METHODS SECTION**

**Changes in leakage management**

Since 2013 EVT became an alternative option for the primary therapy of anastomotic leakage due its clinically evident success and several positive case series [[1-3](#_ENREF_1)]. During the following years, EVT became 'first-line' therapy for treatment of UGI leakages and thus surgical revision or SEMS became secondary choice. We made this decision based on our own experience but also the increasingly available published literature that predominantly [[4-7](#_ENREF_4)], but not exclusively [[8](#_ENREF_8)], demonstrated a higher effectiveness of EVT compared to other endoscopic treatment options such as SEMS [[9](#_ENREF_9)].

During the early years, EVT was performed at the surgical and gastroenterological endoscopic units and up to 15 endoscopists were involved. As EVT became the standard-of-care in leakage management, its successful implementation was prioritized and efforts were made to provide the most experienced endoscopists for these procedures. Thus, the number of specialists involved was reduced and a senior endoscopist responsible for EVT was designated.

We additionally performed routine early postoperative endoscopic investigations in cases of persistent or increasing inflammation parameters even when the patient appeared otherwise normal. The predictive value of CRP to detect postoperative leakages is known [[10](#_ENREF_10)]. The endoscopic evaluation for possible diagnosis and simultaneous therapy of a potential leak was given priority over radiological imaging (such as swallowing or CT of the abdomen), as its specificity and sensitivity is almost 95% [[11](#_ENREF_11)] while not containing a relevant risk for damage of the anastomosis due to applied intraluminal pressure [[12](#_ENREF_12)]. This approach enabled earlier detection and therapy of leakages or pre-emptive treatment in high-risk patients before clinical decompensation or sepsis occurred. The principle and efficacy of pre-emptive EVT therapy has already been described [[13](#_ENREF_13), [14](#_ENREF_14)].

The provision of negative pressure has also undergone several changes. In the first time period, vacuum container (REDYROB®, B. Braun SE, Melsungen, Germany) and then three different electronic pump systems (KCI / 3M Inc., San Antonio, USA; Paul Hartmann AG, Heidenheim, Germany and Medela AG, Baar, Switzerland) were applied. In order to improve applicability, we decided to only use the Medela System during the second period. A negative pressure of 125mmHg was usually applied. In unstable cavities and in the vicinity of sensitive organs/structures, suction was reduced to 80 to 100 mmHg. In large, stable caves, suction was increased to 150mmHg.

Notably, we abandoned the usage of parallel enteral feeding tubes in the vast majority of cases as we found them to prevent the development of sufficient negative pressure treatment when the sponge was placed intra-luminally. Thus, in order to ensure sufficient enteral nutrition, patients with a high risk of leakage (e.g. from oncological esophageal resections) received a percutaneous feeding tube routinely during the primary operation [[15](#_ENREF_15)]. Patients without a percutaneous feeding tube were managed with total parenteral nutrition during EVT.

Finally, patients presenting with small (< 5 mm) residual findings were frequently converted to SEMS or over-the-scope-clip OTSC (Ovesco AG, Germany) therapy to shorten treatment time and to expedite their discharge from hospital.

**Analyzed Variables and definitions**

The prospectively maintained database of EVT patients was expanded to contain additional variables that were retrospectively collected. All data was collected in an anonymized database (Excel 2016, Microsoft, USA) containing several variables without identifying data that would enable patient identification. The following items were ultimately collected: Baseline: Age, gender, comorbidity (including American Association of Anesthesiologists ASA score [[16](#_ENREF_16)] and Charlson Comorbidity Index [[17](#_ENREF_17)], UGI disease, neoadjuvant therapy, type of index procedure/surgery, anastomotic reconstruction, type of leakage, leakage diameter, sepsis on diagnosis of leakage defined according to the international consensus definition [[18](#_ENREF_18)]; primary therapy of leakage. EVT: sponge localization, number of changes, improvement (according to endoscopy and inflammation parameters) and/or resolution (defined as healing of leakage without further interventions after the end of EVT) of leakage during EVT, local complications during EVT, recurrent sepsis after start of EVT, additional procedures for leakage therapy and local infection control. Failure-to-cure defined as in-hospital death or stop of EVT due to complications or missing improvement, with consecutive surgical therapy or switch to SEMS therapy. Postoperative complications graded according to Clavien-Dindo [[19](#_ENREF_19)] and the comprehensive complication index (CCI) [[20](#_ENREF_20)]. MTL30 defined as in-hospital-mortality, transfer (which did not occur) or length-of-stay > 30 days. Level of care during EVT. Oral nutrition at discharge. The following time intervals were individually calculated: time from surgery/ endoscopic procedure until leakage, time from diagnosis of leakage until start of EVT, duration of leakage therapy and length-of stay. The data was locally collected from the hospital information system, internally validated and transferred into the database. The data was transferred anonymously to IBM SPSS Statistics, Version 26, for statistical analysis. All variables were checked for plausibility in detail. Plausible entry errors were corrected; implausible data values were deleted.

SUPPLEMENTARY TABLE 1: Total number of surgical procedures and incidence of postoperative/ anastomotic leakage treated by EVT during study period

|  | 2012 | 2013 | 2014 | 2015 | 2016 | 2017 | 2018 | 2019 | 2020 | 2021 | total | EVT, n (%) |
| --- | --- | --- | --- | --- | --- | --- | --- | --- | --- | --- | --- | --- |
| **Benign disease** | | | | | | | | | | | | |
| Hiatal hernia | 139 | 116 | 96 | 171 | 150 | 171 | 169 | 170 | 150 | 60 | 1,392 | 9 (0.6) |
| Metabolic/ Obesity | 120 | 95 | 101 | 87 | 87 | 115 | 183 | 135 | 90 | 52 | 1,065 | 11 (1.0) |
| Achalasia | 13 | 23 | 35 | 105 | 75 | 98 | 63 | 63 | 56 | 19 | 550 | 6 (1.1) |
| Other | 11 | 14 | 9 | 11 | 13 | 11 | 12 | 11 | 19 | 3 | 114 | 2 (1.8) |
| **total** | **283** | **248** | **241** | **374** | **325** | **395** | **427** | **379** | **315** | **134** | **3,121** | **28 (0.9)** |
| **Malignant disease** | | | | | | | | | | | | |
| Esophagus | 13 | 18 | 11 | 12 | 16 | 13 | 18 | 39 | 37 | 24 | 201 | 37 (18.4) |
| Stomach | 44 | 27 | 46 | 35 | 46 | 49 | 48 | 45 | 73 | 16 | 429 | 25 (5.8) |
| **total** | **57** | **45** | **57** | **47** | **62** | **62** | **66** | **84** | **110** | **40** | **630** | **62 (9.8)** |

Only EVT patients with in-house index operation. Patients with preventive EVT and no evidence of leakage after sponge removal were excluded.

**SUPPLEMENTARY TABLE 2: Individual description of the failure-to-cure cases**

| No. | Period 1 | Period 2 |
| --- | --- | --- |
| 1 | 50y, female, SCC esophagus, esophagectomy after neoadjuvant RCTx, anastomotic leakage of esophagogastrostomy on POD 22, development of esophago-tracheal fistula after 7d of EVT, tracheal stent placement and emergency esophageal diversion, development of aorto-bronchial fistula with rethoracotomy, consecutive ARDS with ECMO, death on POD 90 | 70y, male, AEG 1, esophagectomy after neoadjuvant RCTx, anastomotic leakage of esophagogastrostomy on POD 31, development of esophago-tracheal fistula after 37d of EVT, tracheal stent placement and emergency esophageal diversion, consecutive ARDS with ECMO, death on POD 80 |
| 2 | 62y, male, AEG 1, esophagectomy after neoadjuvant RCTx, anastomotic leakage of esophagogastrostomy on POD 11, endoscopic stent placement, switch to EVT due to stent displacement, development of esophago-tracheal fistula after 2d of EVT, tracheal stent placement and emergency esophageal diversion, discharge on POD 58 | 65y, female, pancreatic cancer, duodenopancreatectomy, anastomotic leakage of gastrojejunostomy on POD 14, start of EVT, relaparotomy with pancraetectomy and anastomotic revision on POD 15, discharge on POD 37 |
| 3 | 74y, female, gastric cancer, partial gastric resection, anastomotic leakage of gastrojejunostomy on POD 15, primary EVT, relaparotomy due to persisting leakage after 2 sponge changes, surgical suture of leakage, discharge on POD 38 | 70y, female, enterothorax with laparoscopic reposition and fundoplication, recurrant enterothorax with fundus necrosis on POD 2, revision with fundus resection and merendino procedure, prophylactic EVT, progressive leakage and sepsis during 4d, emergency esophageal diversion, discharge on POD 48 |
| 4 | 64y, male, AEG 1, esophagectomy, anastomotic leakage of esophagogastrostomy on POD 32, resolution of leakage after 14d of EVT, development of pleural empyema and rethoracotomy, consecutive ARDS with ECMO, death on POD 47 | 79y, female, Boerhaave syndrome with pleural empyema, EVT during 20d without leakage resolution, salvage esophageal diversion, discharge 84d after leakage |
| 5 | 48y, female, perforated anastomotic ulcer 2y after Roux-en-Y gastric bypass, development of gastro-colic fistula during 21d of EVT, laparotomy and surgical revision, discharge 38d after perforation | 56y, male, perforated gastric ulcer, partial gastric resection, anastomotic leakage of gastrojejunostomy on POD 13, long-term ICU therapy, resolution of leakage during 50d EVT, died of pneumonia on POD 83 |
| 6 | 76y, female, endoscopic resection of duodenal adenoma, diagnosis of leakage on the consecutive day, EVT during 8d with persistent leakage, open duodenopancreatectomy, discharge 38d after leakage | 50y, male, chronic pancreatitis, duodenopancreatectomy, relaparotomy for pancreatic fistula, anastomotic leakage of gastrojejunostomy on POD 26, improvement of leakage during 27d EVT, fulminant aspiration during sponge change with fatal ARDS, death on POD 54 |
| 7 | 67y, male, pancreatic cancer, duodenopancreatectomy, anastomotic leakage of gastrojejunostomy on POD 8, surgical revision and start of prophylactic EVT, refractory septic multiorgan failure, death on POD 9 |  |
| 8 | 41y, male, retroperitoneal sarcoma, abdominal compartment resection, postoperative duodenal leakage on POD 12, EVT during 18d with persisting leakage, surgical revision, death on POD 33 |  |
| 9 | 59y, female, gastric cancer, gastrectomy after neoadjuvant chemotherapy, anastomotic leakage of esophagojejunostomy on POD 7, persisting leakage during 42d EVT, switch to stent therapy, discharge on POD 64 |  |
| 10 | 37y, female, enterothorax, laparoscopic reposition and fundoplication, leakage on POD 1, resolution of leakage after 6d of EVT, fatal pulmonary embolism on POD 8 |  |
| 11 | 61y, male, SCC esophagus, esophagectomy after neoadjuvant RCTx, anastomotic leakage of esophagogastrostomy on POD 11, development of esophago-tracheal fistula after 1d of EVT, tracheal stent placement and emergency esophageal diversion, consecutive ARDS with ECMO, death on POD 31 |  |
| 12 | 65y, male, SCC esophagus, esophagectomy after neoadjuvant RCTx, prophylactic EVT, necrosis of gastric conduit, emergency esophageal diversion, long-term ICU therapy, death on POD 84 |  |

Y, years; d, day; SSC, squamous cell carcinoma; RCTx, radio chemotherapy; POD, postoperative day; EVT, endoscopic vacuum therapy; ARDS, acute respiratory distress syndrome; ECMO, extracorporeal membrane oxygenation; AEG, adenocarcinoma of the esophageal-gastric junction

**References**

1. Wedemeyer J, Brangewitz M, Kubicka S, Jackobs S, Winkler M, Neipp M, Klempnauer J, Manns MP, Schneider AS (2010) Management of major postsurgical gastroesophageal intrathoracic leaks with an endoscopic vacuum-assisted closure system. Gastrointestinal endoscopy 71**:**382-386

2. Weidenhagen R, Hartl WH, Gruetzner KU, Eichhorn ME, Spelsberg F, Jauch KW (2010) Anastomotic leakage after esophageal resection: new treatment options by endoluminal vacuum therapy. Ann Thorac Surg 90**:**1674-1681

3. Kuehn F, Schiffmann L, Rau BM, Klar E (2012) Surgical endoscopic vacuum therapy for anastomotic leakage and perforation of the upper gastrointestinal tract. J Gastrointest Surg 16**:**2145-2150

4. Schniewind B, Schafmayer C, Voehrs G, Egberts J, von Schoenfels W, Rose T, Kurdow R, Arlt A, Ellrichmann M, Jurgensen C, Schreiber S, Becker T, Hampe J (2013) Endoscopic endoluminal vacuum therapy is superior to other regimens in managing anastomotic leakage after esophagectomy: a comparative retrospective study. Surg Endosc 27**:**3883-3890

5. Mennigen R, Harting C, Lindner K, Vowinkel T, Rijcken E, Palmes D, Senninger N, Laukoetter MG (2015) Comparison of Endoscopic Vacuum Therapy Versus Stent for Anastomotic Leak After Esophagectomy. J Gastrointest Surg 19**:**1229-1235

6. Hwang JJ, Jeong YS, Park YS, Yoon H, Shin CM, Kim N, Lee DH (2016) Comparison of Endoscopic Vacuum Therapy and Endoscopic Stent Implantation With Self-Expandable Metal Stent in Treating Postsurgical Gastroesophageal Leakage. Medicine (Baltimore) 95**:**e3416

7. Brangewitz M, Voigtlander T, Helfritz FA, Lankisch TO, Winkler M, Klempnauer J, Manns MP, Schneider AS, Wedemeyer J (2013) Endoscopic closure of esophageal intrathoracic leaks: stent versus endoscopic vacuum-assisted closure, a retrospective analysis. Endoscopy 45**:**433-438

8. Berlth F, Bludau M, Plum PS, Herbold T, Christ H, Alakus H, Kleinert R, Bruns CJ, Holscher AH, Chon SH (2019) Self-Expanding Metal Stents Versus Endoscopic Vacuum Therapy in Anastomotic Leak Treatment After Oncologic Gastroesophageal Surgery. J Gastrointest Surg 23**:**67-75

9. Rausa E, Asti E, Aiolfi A, Bianco F, Bonitta G, Bonavina L (2018) Comparison of endoscopic vacuum therapy versus endoscopic stenting for esophageal leaks: systematic review and meta-analysis. Diseases of the esophagus : official journal of the International Society for Diseases of the Esophagus 31

10. Park JK, Kim JJ, Moon SW (2017) C-reactive protein for the early prediction of anastomotic leak after esophagectomy in both neoadjuvant and non-neoadjuvant therapy case: a propensity score matching analysis. J Thorac Dis 9**:**3693-3702

11. Hogan BA, Winter DC, Broe D, Broe P, Lee MJ (2008) Prospective trial comparing contrast swallow, computed tomography and endoscopy to identify anastomotic leak following oesophagogastric surgery. Surg Endosc 22**:**767-771

12. Page RD, Asmat A, McShane J, Russell GN, Pennefather SH (2013) Routine endoscopy to detect anastomotic leakage after esophagectomy. Ann Thorac Surg 95**:**292-298

13. Gubler C, Vetter D, Schmidt HM, Muller PC, Morell B, Raptis D, Gutschow CA (2018) Preemptive endoluminal vacuum therapy to reduce anastomotic leakage after esophagectomy: a game-changing approach? Diseases of the esophagus : official journal of the International Society for Diseases of the Esophagus 32

14. Muller PC, Morell B, Vetter D, Raptis DA, Kapp JR, Gubler C, Gutschow CA (2021) Preemptive Endoluminal Vacuum Therapy to Reduce Morbidity after Minimally Invasive Ivor Lewis Esophagectomy: Including a Novel Grading System For Postoperative Endoscopic Assessment of GI-Anastomoses. Ann Surg

15. Zhuang W, Wu H, Liu H, Huang S, Wu Y, Deng C, Tian D, Zhou Z, Shi R, Chen G, Piessen G, Khaitan PG, Koyanagi K, Ozawa S, Qiao G (2021) Utility of feeding jejunostomy in patients with esophageal cancer undergoing esophagectomy with a high risk of anastomotic leakage. J Gastrointest Oncol 12**:**433-445

16. Owens WD, Felts JA, Spitznagel EL, Jr. (1978) ASA physical status classifications: a study of consistency of ratings. Anesthesiology 49**:**239-243

17. Charlson ME, Pompei P, Ales KL, MacKenzie CR (1987) A new method of classifying prognostic comorbidity in longitudinal studies: development and validation. J Chronic Dis 40**:**373-383

18. Singer M, Deutschman CS, Seymour CW, Shankar-Hari M, Annane D, Bauer M, Bellomo R, Bernard GR, Chiche JD, Coopersmith CM, Hotchkiss RS, Levy MM, Marshall JC, Martin GS, Opal SM, Rubenfeld GD, van der Poll T, Vincent JL, Angus DC (2016) The Third International Consensus Definitions for Sepsis and Septic Shock (Sepsis-3). Jama 315**:**801-810

19. Dindo D, Demartines N, Clavien PA (2004) Classification of surgical complications: a new proposal with evaluation in a cohort of 6336 patients and results of a survey. Ann Surg 240**:**205-213

20. Slankamenac K, Graf R, Barkun J, Puhan MA, Clavien PA (2013) The comprehensive complication index: a novel continuous scale to measure surgical morbidity. Ann Surg 258**:**1-7
